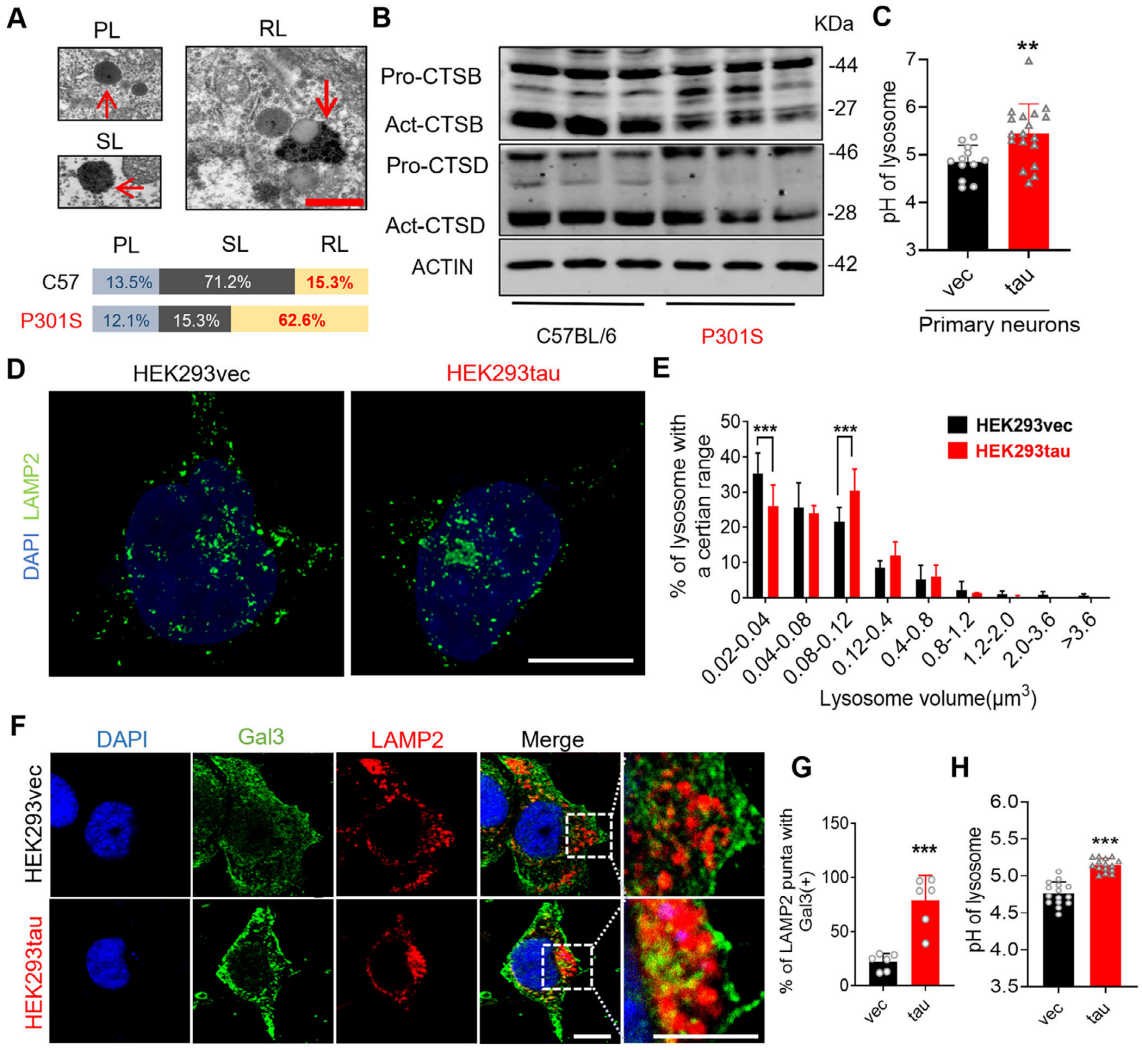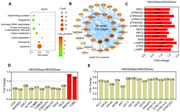# Lysosomal stress response in tau overexpressed cells

**DOI:** 10.1002/alz70861_108034

**Published:** 2025-12-23

**Authors:** Chao‐yuan Ye

**Affiliations:** ^1^ Huazhong University of Science and Technology, Wuhan, Hubei China

## Abstract

**Background:**

Pathological accumulation of microtubule‐associated protein tau and lysosome dysfunction are both important pathological events in Alzheimer‘s disease (AD). It is necessary to study the effects on lysosomes of tau burdened cells systematically.

**Method:**

To get the informations of lysosomes in tau overexpressed cultured cells (HEK293 cells, N2a cells and primary neurons) and brains of mice carrying P301S tau, we used the techniques including proteomics, Western blotting, lysosomal fluorescence imaging, ultramicro‐scopic imaging, and lysosomal functional imaging.

**Result:**

The Lysosome signal was enriched by the differentially expressed proteins in HEK293tau cells, and the disruption of microtubule system and deficiency of lysosomal transporters were also suggested in the proteomic data. The lysosomes in tau burdened cells were larger, less numerous, more perinuclear distributed, deacidified and less active in proteolysis. The number of neuronal residual body type lysosomes (RLs), which contain particles with high electron densities and lipid droplets with low electron densities not been digested, was significantly increased in hippocampi of P301S tau mice.

**Conclusion:**

All these data suggested the lysosomal stress response in tau overexpressed cells, and helped to understand the role of tau in the neurodegeneration of AD.